# Drone Detection and Defense Systems: Survey and a Software-Defined Radio-Based Solution

**DOI:** 10.3390/s22041453

**Published:** 2022-02-14

**Authors:** Florin-Lucian Chiper, Alexandru Martian, Calin Vladeanu, Ion Marghescu, Razvan Craciunescu, Octavian Fratu

**Affiliations:** Telecommunications Department, University Politehnica of Bucharest, 060042 Bucharest, Romania; florin_lucian.chiper@upb.ro (F.-L.C.); calin.vladeanu@upb.ro (C.V.); ion.marghescu@upb.ro (I.M.); razvan.craciunescu@upb.ro (R.C.); octavian.fratu@upb.ro (O.F.)

**Keywords:** drone, UAV, RF methods, software-defined radio, detection system, defense system

## Abstract

With the decrease in the cost and size of drones in recent years, their number has also increased exponentially. As such, the concerns regarding security aspects that are raised by their presence are also becoming more serious. The necessity of designing and implementing systems that are able to detect and provide defense actions against such threats has become apparent. In this paper, we perform a survey regarding the different drone detection and defense systems that were proposed in the literature, based on different types of methods (i.e., radio frequency (RF), acoustical, optical, radar, etc.), with an emphasis on RF-based systems implemented using software-defined radio (SDR) platforms. We have followed the preferred reporting items for systematic reviews and meta-analyses (PRISMA) guidelines in order to provide a concise and thorough presentation of the current status of the subject. In the final part, we also describe our own solution that was designed and implemented in the framework of the DronEnd research project. The DronEnd system is based on RF methods and uses SDR platforms as the main hardware elements.

## 1. Introduction

Technical innovations continue to manifest at an ever-increasing speed, causing fast and drastic changes to modern society. These changes, driven by the possibilities offered by new technologies, affect citizens, governments, and all public and private industry sectors.

As a result, the development of small, low-cost unmanned aerial vehicles (UAVs), commonly known as drones, has resulted in an ever-increasing number of these devices being utilized in a variety of applications [1]. UAVs have introduced new participants in aviation, quickly evolving beyond their military origin to become powerful business tools [2,3].

Applications of UAVs range from recreation to commercial and military applications, including enjoyment, hobbies, games with drones, homemade entertainment videos, recreational movies [4,5,6], low altitude flying base stations [7], and the operation of UAVs for military purposes [8,9,10,11,12,13].

The following research questions were developed for this project:

- What functions should a drone detection and defense system (DDDS) have in order to prove its functionality?
- Which are the most popular methods used in the implementation of DDDSs?
- Which are the main parameters that should be taken into consideration in research?
- What gaps are in the current research of DDDSs?

A widely-used methodology was utilized to conduct a systematic literature review based on preferred reporting items for systematic reviews and meta-analyses (PRISMA) [14] in order to obtain the answers to our study questions. We conducted a literature search in scientific databases that encompass prominent computer science journals and conferences, such as IEEE Xplore, ACM digital library, ScienceDirect, SAGE Journals Online, and Springer Link, to discover key articles on the drone detection and defense systems topic. We used the following search string to discover the relevant publications and papers for our research: (‘Drone’ OR ‘UAV’) AND (Counter) in the domains of electrical engineering, applied physics, telecommunications, defense, and computer information systems, for the previous six years (2016–2021). In total, we gathered a set of 7349 potentially relevant publications, excluding grey literature and pre-prints.

**Figure 1:**
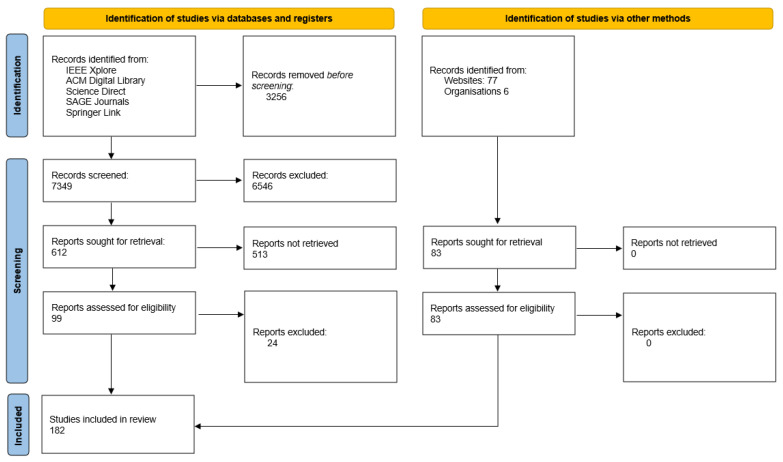
PRISMA 2020 flow diagram for systematic reviews.

We next looked at the titles, keywords, and abstracts of the publications in order to find the papers and articles that described at least one distributed ledger modeling or simulation approach. We chose a total of 99 publications in the process. We examined the references of the selected publications for other papers that were relevant to our inquiry in order to expand our literature collection. Figure 1 shows the overall number of articles produced as a result of this approach.

The additional references that were identified in the bodies of the selected publications, or referencing those, were added to the literature list. We carefully studied the selected publications once the literature selection procedure was completed in order to determine the described applications and problems. The results of our analysis are reported in the following sections, which represent the core of the topical literature review.

The main contributions of our paper can be summarized as follows:

- We provide a detailed review regarding the drone defense systems based on RF methods, focusing on the solutions that are based on software-defined radio (SDR) platforms. To our best knowledge, other reviews that were performed concerning drone defense systems have not detailed that particular category of solutions;
- We discuss the current worldwide status of the legal issues regarding the jamming function, that enables the systems to annihilate the drones after they are detected;
- We present our own solution for an RF-based drone defense system that was designed and implemented within the framework of the DronEnd research project. The system was developed using several SDR platforms and a custom-made mount for dynamically adjusting the orientation of the jamming antenna, which enables the detection, localization, and annihilation of drones in a given monitored area.

The rest of this article is organized as follows: Section 2 reviews the most recent incidents that involved the reckless flying of UAV systems and the regulations taken by different governments and agencies around the world.

Section 3 describes the system requirements of a drone defense system in correlation with their basic mechanism/sensing technologies, considering their advantages and drawbacks. Also, this section highlights the specific models and architectures used in research for drone detection and defense systems. Section 4 details aspects regarding RF-based DDDSs and the use of SDR platforms in such systems. Section 5 contains a discussion regarding the challenges and the future research directions related to DDDSs. In Section 6, a solution for a drone detection and defense system based on SDR platforms, developed by the authors, is proposed and detailed, also highlighting the novel elements that are brought about, compared to the other existing solutions. The last section concludes the paper and includes the future perspectives of this work.

## 2. The Necessity of Drone Detection and Defense Systems: Incidents and Regulations

The drone industry’s rapid rise has outpaced the rules for safe and secure drone operation, making them a symbol of illegal and destructive terror and crimes [15].

Drones have gained attention as a threat to safety and security since their entrance into civilian technology, which has fueled the development of anti-drone (or counter-drone) technologies. Anti-drone systems are designed to protect against drone accidents or terrorism, but they will need to evolve in order to deal with future drone flight systems [16].

UAVs have been used in a variety of military actions. Non-military UAVs have been accused of endangering airplanes, as well as persons and property on the ground. Due to the potential of an ingested drone to quickly damage an aircraft engine [17], safety concerns have been raised. Multiple near-misses and verified collisions have occurred involving hobbyist UAV pilots operating when violating the aviation safety standards [18].

### 2.1. Recently Reported Incidents

The necessity of anti-drone defense systems has gained importance, considering the large number of dangerous occurrences that are mentioned in Table 1.

In addition to the highlighted incidents, the number of small mishaps caused by unauthorized or illegal drones invading restricted regions is increasing by the day [30]. This is another reason for anti-drone technology becoming increasingly important. As the regulations concerning drone usage are also a significant aspect to be considered when designing a DDDS, we review in the following subsection several aspects in this matter.

### 2.2. Regulations Regarding the Use of Drones

The most important agencies that regulate the use of drones (e.g., European Union Aviation Safety Agency (EASA), Federal Communication Commission (FCC), Australian Communication and Media Authority (ACMA), Civil Aviation Authority (CAA), etc.) have adopted action plans in order to ensure critical objectives against the illegal usage of UAVs [30,31,32].

For example, in order to address the hazards and threats posed by drones, European Union members in EASA have endorsed a counter-unmanned aerial systems (counter-UAS) action plan, proposed by the agency in 2019, which has subsequently been included in the European Plan for Aviation Safety (EPAS) [32].

The EASA’s EPAS is applicable to all of the national and appropriate agencies, and it has resulted in the effective control of UAV hazards.

Furthermore, the EU has approved EASA’s standard European guidelines in order to enable UAV integration and safe operation in the aviation system. The rules that apply to drones are outlined in Regulation (EU) 2019/94735 on the rules and procedures for the operation of unmanned aerial vehicles (UAVs) and Regulation (EU) 2019/945 on unmanned aerial vehicles and third-country operators of unmanned aerial vehicles (UAVs).

According to the document, there are three primary types of drone incident offenders that endanger civil aviation, as follows: non-criminal motivation, gross negligence, and criminal/terrorist motivation [30]. They relate to the drone’s remote pilot’s intention, as described in Table 2.

## 3. Drone Detection and Defense Systems: Classification, Sensors, Countermeasures

In this section, we focus on the classification of drone detection and defense systems depending on different criteria, on the comparison of the different sensor types that can be used in order to detect the presence of the drones in the monitored area, on the classification of the countermeasures that can be adopted in order to annihilate the detected drones, and on the regulations regarding the use of jamming as countermeasure.

### 3.1. Classification of Drone Detection and Defense Systems

Firstly, it is necessary to classify DDDSs in order to understand their capabilities, as it is summarized in Table 3.

A DDDS implies different available technologies for detection, tracking, and classification, in addition to neutralization techniques. The most essential elements recommended for the DDDS are considered to be detection, tracking, and classification of the target drones [30,34]. The different technologies that are used for allowing drone detection are summarized in Table 4.

### 3.2. Classification of Detection Sensors

All of the types of sensors that are currently used in DDDS present specific advantages and limitations and, as a direct consequence, such a system must incorporate more sensors of different types in order to achieve a higher detection rate [33].

A brief description of each category of sensors is given below and the different pros and cons for each category are summarized in Table 5.

#### 3.2.1. Radio Frequency Sensors (RF)

UAV RF detection is a technique that involves the interception and analysis of the signals transmitted (Tx, Rx) between the UAV and the ground station. Usually, these signals consist of uplink (from the ground station) control signals and downlink (from the drone) data signals (position and video data) [103]. A detailed analysis of the DDDSs that are based on RF methods are presented in Section 4.

#### 3.2.2. Radar

The Radar solution for drone defense systems represents an active method to identify and localize a potential UAV threat. In order to determine the range, angle, or velocity of a UAV, radar is widely used as an active sensor in sensing systems in a DDDS. A radar system consists of a transmitter, a receiver, and a processor [73].

#### 3.2.3. Imaging Sensors

This technology involves the use of cameras that take images from a designated area in order to determine the presence of a target drone.

##### Electro-Optical (EO) Cameras

Some DDDS use imaging sensors (EO/IR), which could be led by other sensors (such as radar and RF) in order to obtain images of the drone and its main characteristics (e.g., payload). These images can be recorded and analyzed by specialists in order to determine the threat level [55].

The main disadvantage of this method is its low performance under dark and foggy conditions. Moreover, the quality of the images depends on the quality of the lenses and the angle of the photography (LoS is mandatory).

##### Infrared (IR) Cameras/Thermal

This method employs thermal IR cameras that are able to detect the heat produced by a UAV’s hardware components, such as the motors, batteries, and processors. 

This detection method presents disadvantages related to detection range and environment caused by the sensibility of the sensors that measure the thermal difference between the drone and the background. In consequence, the detection of drone presence depends on the drone’s motor temperature, angle (LoS is mandatory), distance, and the temperature of the IR sensors [58].

#### 3.2.4. Acoustic Sensors

This technology involves the use of a microphone array that captures the noise generated by the propellers and rotors of a UAV and compares it with an intern acoustic signature database [42].

Table 5 summarizes the advantages and limitations of each of the different technologies that were mentioned above.

### 3.3. Classification of Countermeasures

The necessity of DDDS arose for the first time in military applications under special regulations that exceed other governmental or structure capabilities and responsibilities. In consequence, the neutralization techniques are more numerous than the detection techniques [30].

The most important DDDS countermeasures are as follows:

- *Electromagnetic pulse (EMP)*—a beam generated with the goal to damage the internal electronics of the target drone [115,116,117];
- *Interceptor drone/Collision Drone*—a drone used to force the target drone to land or return home [118,119,120,121,122,123];
- *Lasers*—directed rays used to destroy the target or blind the camera (dazzler) [124,125,126,127,128,129];
- *Magnetic*—use powerful magnets in order to create a magnetic field around a protected area [130];
- *Prey birds*—eagles or falcons specially trained to attack the enemy’s drone [131];
- *Shooting nets*—a net is launched towards the target drone to prevent the propellers from rotating [132];
- *Projectiles*—large-caliber ammunition used to destroy the target [133];
- *Missiles*—conventional ammunition, could be guided or unguided [133];
- *Guns*—conventional weapons and ammunition [133];
- *Water cannons*—a stream of water is directed towards the target drone [134];
- *RF/GNSS jamming*—disrupt the communication of the target drone with the control station and/or global navigation satellite system (GNSS) [135,136,137,138,139];
- *Spoofing*—decoys the drone by using imitation GNSS and control signals in order to take over the command [140,141,142,143,144,145];
- *Mixed countermeasure techniques*—use two or more countermeasures in order to maximize the neutralization rate.

The main advantages and drawbacks of each of the different countermeasure technique are presented in Table 6.

However, as pointed out in [35], destroying the drone does not mean that the problem is solved. Even if a drone is destroyed using one of the methods listed above, it is just half of the answer. It is critical to discover and detain the operator of the illegally flying UAV in order to resolve the problem completely. Without this, a motivated operator will almost certainly return with a newer and better UAV capable of causing even more disruption and damage.

### 3.4. Regulations Regarding the Use of Jamming in DDDSs

For most of the above-mentioned categories of countermeasures, there are not currently any regulations in force. However, in the case of RF jamming, several existing regulations apply, which will be detailed in the following paragraphs.

The neutralization of drones using jammers is still (in most countries) not legally permitted and is currently the subject of numerous regulatory and legal discussions.

The EU authorities were among the first organizations that took a position regarding the use of jamming devices. The Directive 2014/53/EU prohibits the use of such devices that could cause harmful interferences to the authorized radiocommunications and prevent the normal operation of the communications using radio frequencies [146]. This directive was transposed in all of the member state’s legislations.

The Directive 2014/53/EU was transposed into Romanian legislation by Government Decision no.740/2016. According to this decision, the manufacture, importation, possession, advertising, placing on the market, making available on the market, putting into service and/or use of radio equipment or devices designed to cause harmful interference (jammers) are all prohibited and sanctioned with contravention [147].

In the UK, there were a lot of concerns regarding the collateral damage and the safety risks that must be taken into consideration when using jamming, because of the radio signal interference and the impact on other airspace users. However, only a few regulations have stated that such technology should not be used in any circumstances [148].

The FCC (Federal Communications Commission) in the United States does not merely state that the manufacture, sale, importation, and operation of jammers are all forbidden (Communications Act of 1934, Section 301), but that there are some exceptions, such as institutions under the US government. There is always the risk of a drone losing control, crashing, and causing property damage, or personal harm, when a drone jammer is deployed. This means that anyone using a drone jammer, even government-authorized workers, could be held liable. As a result, the deployment of drone jammers by private entities, such as power companies or airports, is still sporadic but strictly regulated. Only the federal government has the ability to approve the use of drone jammers, and this rigorous restriction extends to their manufacture, importation, and sales [149].

In the Russian Federation, flying a drone is legal. However, most Russian cities are equipped with GPS jammers, which create radio interference, preventing electronics, such as drones, from operating normally. As a consequence, drone users have to keep a safe distance from them because all of the major cities have integrated GPS jammers that can interfere with their drone positioning [150]. Also, there are some regulations that prohibit flying a drone within 500 m of a military installation.

In P. R. China, only the local authorities can use jammer “guns” and other RF DDDSs [151].

Despite of the lack of regulations regarding the use of RF jamming signals against drones, and some risks that should be taken into consideration, this method has to be considered to be among the most efficient.

## 4. Drone Detection and Defense Systems Based on RF Methods

As was mentioned in Section 3, one of the most used methods for drone detection is the identification of the RF signals that are exchanged by the drones with another entity (ground station/operator). Moreover, the annihilation of the detected drones can also be obtained by RF methods, by means of transmitting strong enough jamming signals that can interrupt the communication between the drone and its operator (as mentioned in Section 3.3).

Usually, drones operate on different frequencies, but most commercial drones operate in Industrial, Scientific, and Medical (ISM) frequency bands of 433 MHz and 2.4/5.8 GHz. The simple power detection in these bands will not work due to the presence of other legitimate users in the same geographical area. Therefore, most of the modern RF detection systems provide the detection and identification of the special and unique signals that are generated by the UAV or the data protocols implemented in a UAV.

There are two main functions that are necessary for the detection of the drones, as follows: The *identification* of the presence of the drones by scanning the frequency spectrum and *localization* of the drones. The *annihilation* function, which is necessary in order to allow the defense against the detected drones, can be performed by means of RF jamming, in order to interrupt the communication between the drones and their operators. Table 7 summarizes the main elements regarding the implementation of such systems. In the following paragraphs, each of the below mentioned categories will be detailed.

Most of the RF-based solutions that are described in the literature focus only on the detection of the drones and do not propose countermeasures for the annihilation of the detected drones. One of the reasons behind this might be the increase in the complexity and price of the system that will be generated by the inclusion of such countermeasures in the designed system. A second reason might be related to the fact that most of the references that will be commented on in this section include academic research, in which the target was not the design of a complete commercial system. A third reason could be the fact that jamming equipment is not legal in many areas worldwide, as discussed in Section 2 and Section 3. However, as mentioned previously, the jamming solution can be used in most of the countries if the system that generates it is used for national security or public order purposes. 

Almost all of the implementations that were used for validating the solutions that are proposed in the literature are based on SDR platforms because of some of the significant advantages that are offered by this category of platforms, such as the following:

- Low to moderate cost;
- Extended frequency range, which can usually cover all of the frequency bands that are used by commercial drones;
- Scalability, allowing the extension of the platform, depending on the functions that are foreseen, to be implemented;
- Flexibility, allowing the processing of RF signals corresponding to different communication standards.

Only a few of the existing works include aspects that are related to both of the functions that were mentioned previously as necessary for the detection of the drones, *identification* and *localization*. Such an example is [152], where the authors proposed a drone detection system based on multi-dimensional signal feature identification. After identifying the channel on which the drone communicates with the controller, features, such as signal frequency spectrum (SFS), wavelet energy entropy (WEE), and power spectral entropy (PSE), are extracted in order to allow a precise identification of the drone. In a subsequent step, MUSIC and RAP-MUSIC algorithms are used for performing the localization of the drone, by using information, such as azimuth and elevation. The proposed solution is implemented and tested using USRP X310 SDR platforms and a circular antenna array, obtaining an average detection rate of more than 95%.

In most of the papers that are concerned with the *identification* of the drones RF fingerprinting techniques are used, which rely on the unique characteristics of the RF signal waveforms captured from different drones [153,154,155,156,157]. In [153], a classification of the detected drones is made using a deep residual neural network (DRNN), the results being validated using a USRP X310 SDR platform as a receiver and nine different drones as targets. The authors of [154] separate Wi-Fi and Bluetooth signals from UAV transmitted signals based on their bandwidth and modulation features and classify the UAV signals using machine learning (ML) techniques. In [155], the detection of multiple drones is performed using the k-nearest neighbor (KNN) algorithm after performing a short-time Fourier transform (STFT) on the received signal. A real-time testbed based on the USRP B210 SDR platform is also used for evaluating the performance of the proposed method. A combination of RF fingerprints and hierarchical learning is used in [156] for the classification of the detected drone signals. A Wi-Fi statistical fingerprint approach is proposed in [157], which accounts for the particular characteristics of the Wi-Fi control traffic produced by drones and their remote controllers. 

In [158], a solution that is based on the low cost LimeSDR platform is developed for detecting the presence of drones in the 2.4–2.5 GHz ISM band. The authors use the LMS7002M RF chip from the LimeSDR module but customize the firmware of the FPGA located on the same SDR platform in order to implement the signal processing steps that are necessary for the identification of the RF signals that are transmitted by different drones.

The authors of [159] apply a STFT on the RF signals that are collected using a spectrum analyzer and calculate the time guards associated with the different hopping sequences using the autocorrelation function (ACF) in order to obtain an accurate differentiation of the different UAV remote control (RC) signals.

The following paragraphs will detail the different approaches that were proposed for the implementation of the *localization* function.

A received-signal strength- (RSS-) based 3D localization system utilizing a software-defined radio is proposed in [160], using the recursive least squares (RLS) algorithm in order to numerically estimate the drone’s 3D position.

The authors of [161] propose a localization approach based on the arrays of directional antennas, for obtaining the direction of arrival (DoA) of the NTSC signal that is transmitted by the drones. 

Although the articles that were mentioned above only focused on the detection of drones based on RF methods, there are also papers that present implementations of the annihilation function using RF jamming as a countermeasure against drones [163,164,165]. 

In [162,163], a low-cost SDR platform, BladeRF X40 (Nuand, San Francisco, CA, USA), was used as hardware to implement a jamming system against unauthorized UAVs. The GNU Radio toolkit was used as a software environment for performing the necessary signal processing tasks. In [162], the communication of the drone with the remote control in the 2.4 GHz ISM band was targeted, whereas in [164] the GPS navigation system was targeted.

The authors of [164] implemented a protocol-aware jammer using the BladeRF SDR platform as hardware. Tests were made to target the Futaba Advanced Spread Spectrum Technology (FASST) and the Advanced Continuous Channel Shifting Technology (ACCST) UAV remote control systems.

In [165], a portable jammer is proposed, based on the HackRF One SDR platform and a Raspberry Pi as a host computer. Multiple tests were made in order to validate the proposed solution, in both the 2.4 GHz and 5.8 GHz ISM bands and in the GPS L1 band.

## 5. Challenges and Future Perspectives for Drone Detection and Defense Systems

The previous sections contained a review of the different approaches that can be used for implementing a DDDS. In this section, the challenges that currently have to be faced when developing such a system will be detailed, together with a discussion regarding the future perspectives of this domain.

One of the challenges that is faced when implementing a DDDS is the ability to identify and, in a further step, to annihilate not only one, but several different target drones. In recent years, many applications have used multiple drones [166], therefore, such a feature becomes an important characteristic for a DDDS. Depending on the sensors that are used in the system, the possibility of detecting several target drones may or may not exist. A few examples of systems that include such a feature exist in the literature. In [167,168], algorithms are developed in order to allow multi-UAV detection using video streams. In [169], an RF-based deep learning (DL) algorithm is proposed for performing multiple drone detection. The possibility of a simultaneous annihilation of several drones is an even more challenging task. Electromagnetic pulses (EMP) have been proposed as a possible solution for defense against drone swarms [170]. RF jamming performed using antenna arrays could also generate, by means of signal processing methods (beamforming), multiple beams that could be targeted towards multiple target drones.

Another challenge that a DDDS would have to face, especially if the area in which the system is installed is a residential area, and there are several households in the close neighborhood, is to avoid interference or damage to nearby equipment (in the case of RF jamming and EMP) and to respect the privacy of the nearby neighbors (in the case of imaging sensors). In the case of RF jamming, this could be solved if the antennas that are used or the beams, in the case of using a beamforming approach, are very directive and targeted directly towards the target drone(s).

When referring to a DDDSs based on RF methods, one of the main challenges that has to be addressed is related to the legal issues around the use of jamming as a countermeasure, as was also commented on in Section 3.4. For the time being, in most of the regions worldwide, such a countermeasure can only be legally used when it is integrated into a system that is used for the defense of national security or for public order objectives. However, as the number of situations when such a system would be necessary also applies to the defense of private areas that cannot be included in the above mentioned categories, it is to be expected that the legislation in this domain might be modified in the near future in order to include the possibility of private users also legally using such a system, as long as the interference caused to the nearby areas is kept below certain well-defined thresholds.

An important limitation of RF-based DDDSs is related to the impossibility of detecting and annihilating autonomous drones in cases when they have a predefined flying path and do not have any active data communication with an operator located on the ground.

As mentioned in Table 5, each of the different types of sensors (RF, radar, imaging, and acoustic) has its own drawbacks and limitations. As such, the performance of a DDDS that is implemented using a single type of sensor is directly affected by the disadvantages and limitations of that particular category. By combining several different sensor types in a single *hybrid DDDS*, the system could benefit from the advantages of each different category of sensors. A first benefit would be the increase in accuracy that such a hybrid system could achieve, when the information regarding the identification and localization of the drone would be obtained from multiple different sensors. A second benefit would be related to the possibility of detecting the target drone in situations when one of the sensor types would not allow the detection on its own. For example, if we consider a hybrid DSSS that is implemented using both RF and imaging sensors, the imaging sensors could be used for detecting autonomous drones (that cannot be identified using the RF sensors) and the RF sensors could be used for detecting drones in low visibility conditions (when the imaging sensors could not provide the detection). Very few implementations of such hybrid systems are described in the literature (for example those in [105,115]), and we consider that such an approach is a promising future research and development direction for DDDSs. 

## 6. DronEnd Detection and Defense System

In the current section, a drone detection and defense system, designed and implemented by the authors, together with a research team from the cybersecurity company Cyberwall [171], will be presented. The system was developed within the framework of the DronEnd research project [172]. The preliminary details regarding the project were given in [173].

**Figure 2:**
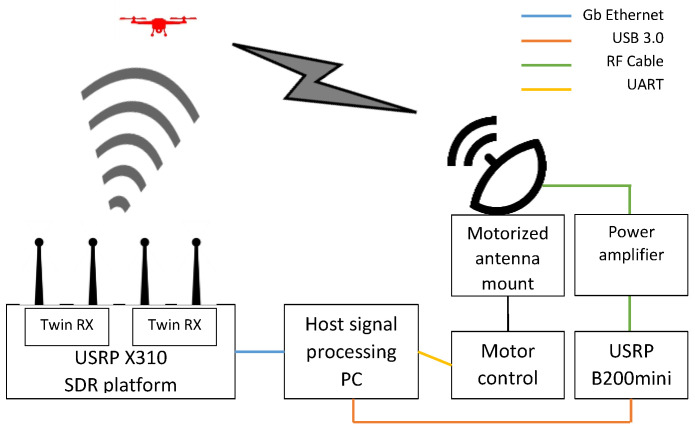
Block diagram of the DronEnd ground defense system.

The goal of the DronEnd ground defense system is to secure a certain area against the unauthorized presence of drones. In order to achieve this goal, the DronEnd system scans the RF spectrum in order to detect the presence of the drones in the supervised area, identifies the location of the drone by means of AoA algorithms, and annihilates the drone by using RF jamming methods. The block diagram of the implemented DronEnd ground defense system is presented in Figure 2.

In the following subsections, all of the elements of the system will be detailed, highlighting the steps that are necessary in order to perform the functions of detection, localization, and annihilation of the drone through jamming.

### 6.1. Detecting the Presence of the Drone Using Spectrum Sensing Algorithms

A first step required for detecting the presence of a drone in the case of RF-based drone defense systems is to monitor the radio spectrum through a spectrum sensing process in order to identify the signals that are transmitted by the drone. For the implementation of the spectrum sensing process in the DronEnd system, spectrum sensing algorithms based on the energy detection method have been used. Algorithms, such as 3EED [174] and 3EED with an adaptive threshold [175], that were previously developed, provide improved performance compared to the classical energy detection (CED) [176] algorithm and were used to identify the presence of the drones in the monitored area. The above-mentioned algorithms were implemented on SDR platforms from the USRP family (USRP X310 (Ettus Research, Santa Clara, CA, USA) [177] equipped with Twin-RX RF Daugterboards (Ettus Research, Santa Clara, CA, USA) [178], 10–6000 MHz frequency range). The frequency bands that are used by the drones that were used to test the DronEnd system (DJI Mavic Air (SZ DJI Technology Co., Ltd., Shenzhen, China) [179], DJI Phantom 4 Pro v2.0 (SZ DJI Technology Co., Ltd., Shenzhen, China) [180], and DJI Mini 2 (SZ DJI Technology Co., Ltd., Shenzhen, China) [181]) were the 2.4 GHz (2400–2500 MHz) and the 5 GHz (5730–5830 MHz) ISM bands, which can be covered using the above-mentioned SDR platforms that can receive signals on frequencies up to 6 GHz. Because the position of the target drones was not initially known, omnidirectional antennas were used in this step.

**Figure 3:**
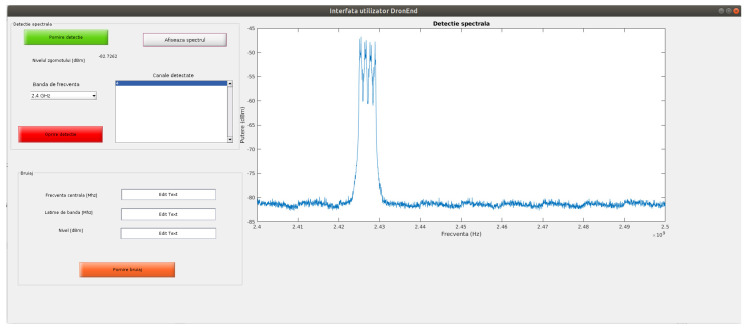
Graphical user interface of the spectrum sensing process implemented in the DronEnd system, showing the signal transmitted by the DJI Mavic Air drone in the 4th channel of the 2.4 GHz ISM band.

Figure 3 shows the graphical user interface that was implemented in order to view the results of the spectrum sensing. The signal that was transmitted by the DJI Mavic Air drone on channel four of the ISM 2.4 GHz band can be seen as captured using the USRP X310 SDR platform. In the following subsections, the other elements of the DronEnd system will be detailed, highlighting the steps that are necessary in order to perform the functions of localization and annihilation of the drone through jamming. The capture of the RF data was performed using a GNU Radio python script. As the instantaneous bandwidth captured using the Twin-RX RF daughterboard is smaller than 100 MHz, in order to cover the 100 MHz bandwidth of the 2.4 GHz and 5 GHz ISM bands, several sub-bands were concatenated.

Once the signal that is transmitted by the target drone is detected, the next step is triggered, which is to localize the angle of arrival of the received signal, as will be discussed in the next subsection.

### 6.2. Localization of the Drone Using AoA Algorithms

**Figure 4:**
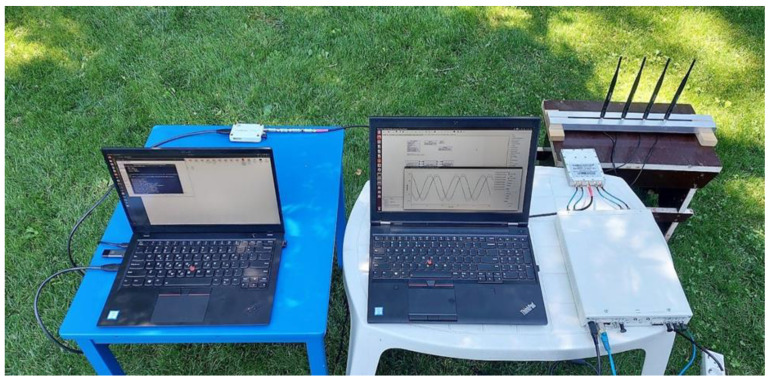
The linear antenna system that was used and the USRP X310 SDR platform during the calibration procedure of the Twin-RX RF modules.

Once the frequency that is used by the drone to communicate has been identified, a second necessary step is to obtain information about the position of the drone. This step was performed using AoA algorithms for detecting the angle of incidence of the detected RF signal. Such algorithms exploit the phase difference of the signals that are received from the drone using a multi-antenna system. The SDR platform that was used as the hardware for providing the RF receive front-end was the USRP X310 [177], on which two Twin-RX RF modules [178] were mounted (covered frequency range of 10–6000 MHz, instantaneous bandwidth 80 MHz). Each of the Twin-RX modules offers two coherent reception channels, and the local oscillator that was used can be shared by the two boards, so that in the end, a total of four coherent reception channels are obtained and are aligned in phase. The antenna system that was used was a linear system of four antennas, spaced at a distance equal to half the wavelength of the minimum frequency that the drones used for testing could transmit (2.4 GHz). In order to estimate the initial phase difference between the four reception channels, a calibration step was required after each system restart, which involves the transmission of a test signal that will be received through the RF cables of equal length on all four of the reception channels. A 5-port RF splitter (Mini-Circuits ZN4PD1-63HP-S+ (Mini-Circuits, New York, USA) [182]) was used in order to distribute the signals. Figure 4 shows both the antenna system that was used and the USRP X310 SDR platform during the calibration stage.

**Figure 5:**
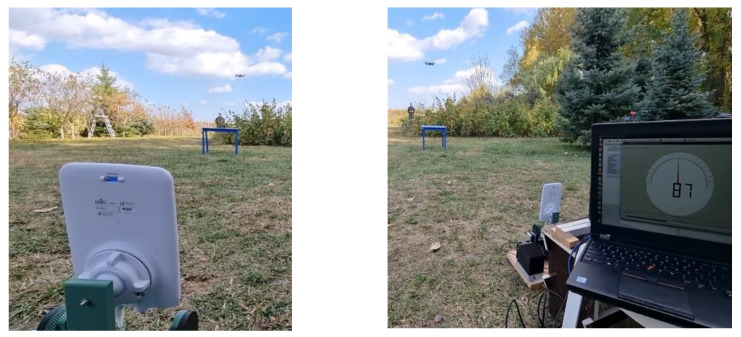
Tests performed using the DronEnd ground system (DJI Mavic AIR target drone). The estimated angle of incidence can be noticed.

Once the calibration step was completed, the four dipole antennas (VERT2450 [183]) that make up the antenna system were connected to the four receive channels of the USRP X310 SDR platform and, based on the phase difference of the signals that were received, the angle of incidence that corresponds to the drone location could be identified by using AoA algorithms. We used one of the classical AoA algorithms, the MUSIC algorithm, and the result was both displayed on a graphical user interface, as shown in Figure 5, and forwarded as an input to the software module that is responsible for setting the orientation of the jamming antenna, which will be detailed in the next subsection.

The positioning that was thus obtained was one in azimuth, as the antenna system that was used was placed horizontally. By using a second system that was located in a vertical plane, the elevation of the drone could be also estimated.

### 6.3. Annihilation of the Drone Using RF Jamming

**Figure 6:**
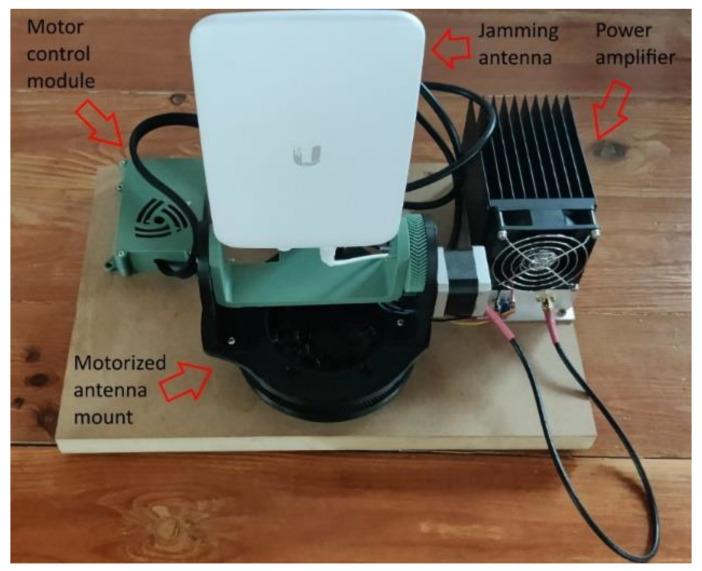
Components used for transmitting the jamming signal.

A final step is to transmit a jamming signal to the identified target drone in order to disrupt the communication between the drone and its operator. As the jamming signal should only be transmitted in the direction of the target drone, in order to avoid interference with other equipment in the area, a directional antenna was used for the jamming operation. Figure 6 shows the following components that were used to implement this step: the transmitting antenna, the motorized antenna mount, the stepper motor control module that was used to move the antenna mount, and the power amplifier.

The angle of incidence that was detected by the AoA algorithm was processed (filtered) using a script implemented in the Matlab environment in order to remove any erroneous indications related to the position of the drone and was subsequently transmitted using a serial interface (UART) to the motor control module (MCM), which controls the stepper motors that are used to move the motorized support for positioning the jamming antenna. The MCM was based on a Microchip ATMega328p processor, which, using the angle information that is obtained using the AoA algorithm, controls the stepper motors. Two Nema 17 stepper motors, controlled using Texas instruments DRV8825 drivers, were used; one to adjust the azimuth and one to adjust the elevation of the jamming signal antenna. In the current configuration, given that the drone’s position was estimated only in the azimuth plane, the commands were transmitted only to one of the two motors (the one that was responsible for the azimuth movement).

**Figure 7:**
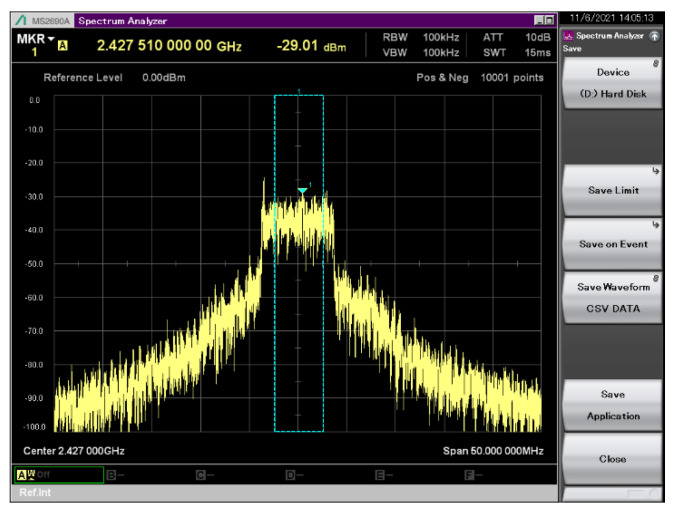
Jamming signal transmitted by the DronEnd ground system on channel 4 in the 2.4–2.5 GHz ISM band.

The SDR platform that was used to generate the jamming signal was a USRP B200mini platform (70–6000 MHz frequency range) (Ettus Research, Santa Clara, CA, USA) [184]. Given that the maximum power that can be obtained at the output of the SDR platform is 10 dBm, a power amplifier (Mini-Circuits ZHL-2W-63-S+ (Mini-Circuits, New York, NY, USA) [185]) was used to amplify the jamming signal in order to extend the range of the system, which offers a 42 dB gain and a maximum output power of 2 W. The antenna that was used to transmit the jamming signal was a Ubiquiti UMA-D (Ubiquiti Inc., New York, NY, USA) directional antenna [186], which covers the 2.4–2.5 GHz and 5.1–5.9 GHz bands and offers a 10 dBi gain in the 2.4 GHz band and a 15 dBi gain in the band of 5.8 GHz. By using a directional antenna that targets the location of the drone for the transmission of the jamming signal, the interferences that are caused to other communication systems that are operating in the neighborhood are minimized. Moreover, the transmit gain can be adjusted depending on the size of the area that has to be protected. Figure 7 shows the jamming signal with a 10 MHz bandwidth emitted in channel four of the 2.4–2.5 GHz ISM band, captured using an Anritsu MS2690A (Anritsu Corporation, Atsugi, Japan) spectrum analyzer.

The tests were performed in an outdoor suburban scenario using the DJI Mavic Air, the DJI Phantom 4 Pro v2.0, and the DJI Mini 2 drones as targets and the annihilation of the drone, which resulted in a forced landing on the position where the drone was located when the jamming signal was turned on, was possible for distances of 40 m from the area where the DronEnd ground system was located.

### 6.4. Conclusion and Future Research Directions

To conclude, the main novel elements that are introduced by the DronEnd system, when compared to other drone detection and defense systems based on RF methods that were mentioned in Section 4, can be summarized as follows:

- Incorporates all of the three functions (identification, localization, and annihilation) that are necessary for a drone detection and defense system in an integrated and scalable platform, which can be reconfigured depending on the requirements of different use cases;
- Includes an agile and accurate identification subsystem, based on improved spectrum sensing algorithms, which performs a real-time identification of the signals that are transmitted by the drone and, moreover, allows a dynamic tracking of the signal transmitted by the drone, even when the transmit frequency is changed;
- Annihilates the detected drone by means of jamming, avoiding at the same time significant interference with nearby devices, as a directional antenna, targeted directly towards the target drone using a motorized antenna mount, is used.

Several aspects are considered as future research directions, in order to improve the performance of the proposed system.

The first direction is related to the possibility of replacing the mechanical motorized antenna mount that was used for targeting the directional jamming antenna with an equivalent static planar antenna array. By using such an approach, the orientation of the resulting antenna beam that was necessary for following the target drone would not involve any moving parts, as the steering would be obtained only by using signal processing methods. The advantages of such an approach would include a smaller delay, the possibility of adjusting the beamwidth by signal processing means, depending on the application necessity, and the absence of aging effects that might affect mechanical parts. However, as the transmit power level that is needed in order to obtain a large enough range for the system might be high, a challenge that would have to be addressed is the design of a power amplification stage for supplying the planar antenna array.

The second direction is related to the addition of a second antenna array, in an orthogonal plane, compared to the one in which the current antenna array is located. By using such a setup, the identification of the target drone could be performed both in azimuth and elevation, allowing for a more precise steering of the directional antenna that is used for transmitting the jamming signal.

The third research direction is related to a subject that was also commented on in Section 5, which is the implementation of a hybrid DDDS in order to improve the overall performance of the system. The addition of imaging sensors is considered, as such an approach would have a twofold contribution; it would improve the accuracy of the detected targets for the situations in which the target drone would be detected by both types of sensors, and it would allow the detection of the target drones also in the situations when only one type of sensor would be able to identify them.

## 7. Conclusions and Future Work

In this paper, a survey related to the current status of drone detection and defense systems was performed and our own solution for a drone defense system based on SDR platforms (DronEnd) was presented. Different aspects, such as regulatory issues and reported incidents that involved drones, were included in the survey. A classification of the drone detection systems that were based on the type of sensors that are used was performed. A detailed description of the RF-based drone detection and defense systems was made, with an emphasis on the use of SDR platforms for the implementation of such systems. The drone defense system that was developed by the authors within the framework of the DronEnd research project is presented in the final part of the paper. As future work, we intend to conduct a detailed testing of the DronEnd ground system, in order to verify the performance of our solution from the detection, localization, and annihilation points of view and we also plan to develop a flying version of the DronEnd system, by mounting an embedded SDR platform on a support drone and approaching the target drones from the air.

## Figures and Tables

**Table 1:** List of the recent UAV-related incidents.

Incident Type	Time and Place of the Event	Short Description of the Incident	Aftermaths	References
Aircraft collisions	17 April 2016/UK, London, Heathrow International Airport	An Airbus A320 collided with a Metropolitan Police UAV as it approached landing	There were no serious issues reported.	[19]
	21 September 2017/USA, Staten Island, New York City	A civilian UAV collided with a Black Hawk helicopter	The helicopter was able to continue flying and landed in a safe manner.	[20]
	12 October 2017/Canada, Jean Lesage Airport, Quebec City	A Skyjet Aviation Beech King Air A100 collided with a UAV	The plane landed safely, with only minor damage to its wings.	[21]
	13 December 2018/Mexico, Tijuana International Airport	On a Boeing 737–800 operating as Flight 773, a “quite loud noise” was heard	After a safe landing, the aircraft’s nose was discovered to be damaged. The reason for the incident has not been identified; however, it was examined as a drone strike by the airline.	[22]
	10 August 2021/UK, Buttonville Municipal Airport	A Cessna 172 registered C-GKWL collided with a drone operated by the York Regional Police	The Cessna landed safely but with significant damage.	[23]
Near-miss incidents	January 2017/P.R. China, Hangzhou Xiaoshan International Airport	A 23-year-old Xiaoshan UAV operator was arrested after taking footage with a drone that flew too close to planes landing	DJI, China’s biggest drone manufacturer and the producer of the Mavic Pro drone (which was discovered to have been used in the event), issued a statement expressing its “strong condemnation” of the illegal filming.	[24]
	25 March 2018/New Zeeland, Auckland Airport	A UAV approached within 5 m of an Air New Zealand Boeing 777–200 on final approach to airport	The pilots spotted the UAV as the plane was approaching a position when evasive action was impossible, and they initially worried it would be pulled into an engine.	[25]
	19 December 2018/UK, Gatwick	A repeated deliberate intrusion of UAVs of “industrial standards” occurred	The suspension of all takeoffs and landings began at 9:03 p.m. on 19 December due to UAV sightings over the runway. Flights were briefly restarted the next morning but were banned again after more UAV sightings.	[26]
Other incidents that targeted officials and strategic objectives	April 2015/Japan	A small drone carrying radioactive materials was dropped on the roof of Japan’s Prime Minister’s mansion	The drone was not only able to fly to the Prime Minister’s home, but it was also left unattended for over two weeks. Due to the characteristics of the area, notably privacy, it may have been difficult to deploy intensive detecting technology.	[27]
	October 2016/Syria	ISIL used two ultra-small drones purchased from Amazon to assassinate two Iranians in Syria	The first incidence of commercial drone terrorism, significant since commercial off-the-shelf drones were employed, demonstrating that a wide variety of drone terrorism was achievable because the drones could be cheaply bought without having the expert-level skill to fly.	[28]
	August 2018/Venezuela	Two bomb-carrying drones had a failed attempt to assassinate Venezuelan President Nicolas Maduro during a national outdoor celebration	The first time a drone was used to try to assassinate the country’s leader. This incident emphasizes the importance of anti-drone technology for avoiding a traumatic event. Temporary anti-drone systems require rapid installation and deployment.	[29]

**Table 2:** EASA categorization of intention/motivation of pilots of unauthorized drones.

Negligence	Individuals Who Are Oblivious to or Are Unaware of the Appropriate Regulations and Constraints. As a Result, They Fly Their Drones across Sensitive or Forbidden Terrain. They Have a “Clueless” Mentality and Have No Intention of Disrupting Regular Aviation.
Gross negligence	Individuals who are reckless because they are aware of the appropriate regulations and constraints yet choose to break them for personal or professional advantage (e.g., aggressive spotters). Their actions can be described as “reckless”, as they disrupt civil aviation while completely ignoring the implications of their conduct.
	Individuals who intentionally strive to use drones to disrupt aerodromes and flight operations, regardless of whether they are aware of the applicable legislation and limits. These individuals may even act as a group to maximize their impact. While their actions may have unexpected repercussions for aviation safety, they do not seek to put human lives in jeopardy.
Criminal/terrorist motivation	Criminals and terrorists are persons who intentionally strive to utilize drones to interfere with the safety and security of civil aviation, regardless of whether they are aware of the applicable legislation and limits. These persons should be considered criminally motivated or even terrorists because their actions are purposeful and show no concern for human lives and property.

**Table 3:** Classification of DDDSs.

Category	Definition
Ground-based: fixed	Systems designed for usage in fixed locations [33]
Ground-based: mobile	Systems designed to be installed on automobiles and operated while they are in motion [33]
Hand-held	Systems designed to be operated by a single person using their hands; the majority of these systems resemble rifles [34]
UAV-based	Systems designed to be mounted on unmanned aerial vehicles (UAVs) [34]
UAV-swarm-based	Systems designed to use multiple drones [35]

**Table 4:** Technologies used for drone detection in DDDSs.

Technology	Description	References
Acoustic	UAVs are detected and tracked by using an array of microphones	[36,37,38,39,40,41,42,43,44,45,46,47,48,49,50,51,52,53]
Imaging (EO/IR)	UAVs are detected and tracked by using EO/IR cameras	[54,55,56,57,58,59,60,61,62,63,64,65,66,67,68,69,70,71,72]
Radar	UAVs are detected and tracked using their radar signature	[73,74,75,76,77,78,79,80,81,82,83,84,85,86,87,88,89,90,91,92,93,94,95,96,97,98,99,100,101,102]
Radio frequency (RF)	UAVs are detected, tracked, and identified by monitoring the radio frequencies used for communications; this technology could localize the UAV and the pilot	[103,104,105,106,107,108,109,110,111,112,113]
Hybrid	Combination of two or more of the above-mentioned technologies	[104,114]

**Table 5:** Pros and cons of sensors used in DDDSs.

Type	Pros	Cons	References
Acoustic	Covers the spectrum of 20 Hz–20 kHz; Acoustic signature library could be updated easily from flight to flight; Lightweight and can be easily associated with other types of sensors.	Limited range; Vulnerable to ambient noise; Susceptible to decoys.	[36,37,38,39,40,41,42,43,44,45,46,47,48,49,50,51,52,53]
Imaging	Covers all of the visible and IR spectrum (3 MHz–300 GHz); IR cameras could operate in cloudy weather and in day or night; Could be assisted by computer-vision technologies.	Provides 2D images; Limited performances by weather conditions and background temperature; Dependent of georeference data LoS is required.	[54,55,56,57,58,59,60,61,62,63,64,65,66,67,68,69,70,71,72]
Radar	Bandwidth used: 3 MHz–300 GHz; Could operate in all weather and day/night conditions; Offers information regarding the velocity of the target; Can recognize micro-Doppler signatures (MDS) Offers high coverage; Good accuracy; Compact and high mobile, required for tactical applications; High reliability.	Large radar cross-section is desired; Difficult to differentiate UAVs from birds; Limited performance for low altitudes and speeds (death cone); Could interfere easily with small objects, especially birds; LoS is required; High cost.	[73,74,75,76,77,78,79,80,81,82,83,84,85,86,87,88,89,90,91,92,93,94,95,96,97,98,99,100,101,102]
RF	Capturing the communication spectrum and signals UAV and operators; Low complexity and easy to implement; Could operate in all weather and day/night conditions; Easier to improve due to modular implementation of receivers and digital signal processing units used in implementation; Possibility to localize the pilot.	Knowledge regarding UAV communication specifications (e.g., frequency bands, modulations, etc.) is required; Difficult to accurately determine AoA; Difficult to use in urban areas due to fading and multipath phenomena; Vulnerable to malicious or illegal modified RF that will exceed receiver capabilities.	[103,104,105,106,107,108,109,110,111,112,113]

**Table 6:** Characteristics and limitations of countermeasure techniques.

Type	Pros	Cons	References
Electromagnetic pulse (EMP)	Could burn or interfere with the internal electronics of the drone, disrupting its operation; Could operate in both narrowband and wideband domains.	Accurate direction of jamming is necessary; Difficult to know the effectiveness of jamming.	[115,116,117]
Interceptor drones	Searching and tracking capabilities; Could carry weapons and ammunition.	Requires a relatively close approach to the target; Have a considerable delay.	[118,119,120,121,122,123]
Lasers	Could operate at low powers (dazzlers) to blind the UAVs cameras or high power, which could burn/destroy the target; Easy to track the target; Cheaper and safer than projectiles or another physical countermeasure.	Sensitive to weather conditions; It is necessary to have an accurate measurement of the target’s position; High power lasers could interfere with other systems.	[124,125,126,127,128,129]
Magnetic	Cost effective; Could respond to multiple threats.	Small protected area; Could interfere with other systems.	[130]
Prey birds	Does not require complex technology; Fewer humans are required.	Applicable only to slower and small UAVs; Could harm the falcons.	[130]
Projectiles/ shooting nets/ water cannons	Effective against any type of UAV; Work in all weather conditions; Quick reaction method.	Might cause collateral damage; High costs; Requires professional operators.	[131,132,133,134]
RF/GNSS jamming	Could neutralize grouped targets simultaneously, degrading their received signal-to-noise ratio (SNR); GNSS frequencies and bands are widely known and relatively easy to jam; The directivity diagram of the jamming signal can be oriented and directed as desired.	Ineffective against autonomous UAVs; Ineffective against drones that use inertial navigation systems/sensors (INS); Ineffective against UAVs that use encrypted communications; Effective only for short distances; The jamming could interfere with other sensible equipment.	[135,136,137,138,139]
Spoofing	DSP and AI algorithms could copy and reproduce the control communication signal with high accuracy in a relatively short time; Could exploit the vulnerabilities of various systems of UAVs.	It is necessary to have a consistent analysis of the targeted UAVs regarding their operation frequencies; Spectrum sensing systems are desirable.	[140,141,142,143,144,145]

**Table 7:** RF-based drone detection and defense systems.

References	Implemented Functions	Methods	SDR Platform Used (Including Manufacturer, City and Country)
[152]	Identification Localization	RF fingerprinting (SFS, WEE, PSE) AoA (MUSIC, RAP MUSIC)	USRP-X310 (Ettus Research, Santa Clara, CA, USA)
[153]	Identification	RF fingerprinting (DRNN)	USRP-X310 (Ettus Research, Santa Clara, CA, USA)
[154]	Identification	RF fingerprinting (CNN)	USRP-X310 (Ettus Research, Santa Clara, CA, USA)
[155]	Identification	RF fingerprinting (KNN)	USRP-B210 (Ettus Research, Santa Clara, CA, USA)
[156]	Identification	RF fingerprinting (KNN, XGBoost)	-
[157]	Identification	RF fingerprinting (Wi-Fi)	-
[158]	Identification	RF fingerprinting	LimeSDR (Lime Microsystems, Guilford, UK)(customized)
[159]	Identification	RF fingerprinting	-
[160]	Localization	Received-signal strength (RSS)	USRP N210 (Ettus Research, Santa Clara, CA, USA)
[161]	Localization	RSS	AD-FMCOMMS5-EBZ Evaluation Board (Analog Devices, Wilmington, DC, USA)
[162,163,164]	Annihilation	RF jamming	BladeRF (Nuand, San Francisco, CA, USA)
[165]	Annihilation	RF jamming	Great Scott Gadgets HackRF One

## Abbreviations

ACCST	Advanced Continuous Channel Shifting Technology
ACF	Autocorrelation Function
ACMA	Australian Communication and Media Authority
AI	Artificial Intelligence
AoA	Angle of Arrival
CNN	Convolutional Neural Network
DDDS	Drone Detection and Defense Systems
DoA	Direction of Arrival
DRNN	Deep Residual Neural Network
DSP	Digital Signal Processing
DL	Deep Learning
EASA	European Union Aviation Safety
EMP	Electromagnetic Pulses
EO	Electro-optical
FASST	Futaba Advanced Spread Spectrum Technology
FCC	Federal Communications Commission
FPGA	Field-Programmable Gate Array
GNSS	Global Navigation Satellite System
GPS	Global Positioning System
INS	Inertial Navigation Systems/Sensors
IR	Infrared
IS	Islamic State
ISM	Industrial, Scientific, and Medical
KNN	K-Nearest Neighbor
LoS	Line of Sight
MDS	Micro-Doppler Signatures
MCM	Motor Control Module
ML	Machine Learning
MUSIC	MUltiple SIgnal Classification
NTSC	National Television Standards Committee
PSE	Power Spectral Entropy
RC	Remote Control
RF	Radio Frequency
RLS	Recursive Least Squares
RSS	Received-Signal Strength
SDR	Software-Defined Radio
SFS	Signal Frequency Spectrum
SNR	Signal-to-Noise Ratio
STFT	Short-Time Fourier Transform
UAS	Unmanned Aerial Systems
UAV	Unmanned Air Vehicle
WEE	Wavelet Energy Entropy
